# Structural Changes of Hierarchically Nanoporous Organosilica/Silica Hybrid Materials by Pseudomorphic Transformation

**DOI:** 10.1002/chem.202000512

**Published:** 2020-06-11

**Authors:** Malina Bilo, Maximilian Münzner, Christian Küster, Dirk Enke, Young Joo Lee, Michael Fröba

**Affiliations:** ^1^ Institute of Inorganic and Applied Chemistry University of Hamburg Martin-Luther-King-Platz 6 20146 Hamburg Germany; ^2^ Institute of Chemical Technology University of Leipzig Linnéstraße 3 04103 Leipzig Germany

**Keywords:** hierarchical porosity, mesoporous materials, pseudomorphic transformation, silicates, solid state NMR spectroscopy

## Abstract

Herein, it is reported how pseudomorphic transformation of divinylbenzene (DVB)‐bridged organosilica@controlled pore glasses (CPG) offers the possibility to generate hierarchically porous organosilica/silica hybrid materials. CPG is utilized to provide granular shape/size and macroporosity and the macropores of the CPG is impregnated with organosilica phase, forming hybrid system. By subsequent pseudomorphic transformation, an ordered mesopore phase is generated while maintaining the granular shape and macroporosity of the CPG. Surface areas and mesopore sizes in the hierarchical structure are tunable by the choice of the surfactant and transformation time. Two‐dimensional magic angle spinning (MAS) NMR spectroscopy demonstrated that micellar‐templating affects both organosilica and silica phases and pseudomorphic transformation induces phase transition. A double‐layer structure of separate organosilica and silica layers is established for the impregnated material, while a single monophase consisting of randomly distributed T and Q silicon species at the molecular level is identified for the pseudomorphic transformed materials.

## Graphical abstract

**A hierarchically nanoporous organosilica/silica hybrid material** was synthesized by impregnation of controlled pore glass (CPG) with divinylbenzene (DVB)‐bridged organosilica and subsequent pseudomorphic transformation. MAS NMR spectroscopy revealed that structural transition is induced by pseudomorphic transformation from a double‐layer structure of impregnated system to a single monophase containing mesopores composed of randomly distributed T and Q Si.

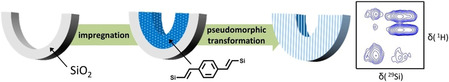

## Introduction

Controlled pore glasses (CPGs) and ordered mesoporous silicas, which are formed by the liquid‐crystal template mechanism and often referred to as micellar‐templated silicas (MTS), are two classes of porous silica‐based materials that are both established in numerous fields of applications, for example, catalysis,^1^, ^2^ chromatography,^3^, ^4^ gas adsorption^5^, ^6^ or enzyme immobilization.^7^, ^8^ CPGs can be synthesized in versatile shapes from monoliths to granules in the micrometer range. Thermally induced phase separation in combination with selective leaching of alkali borosilicate glasses yields a monomodal sponge‐like pore system with narrow pore size distribution adjustable between 4–1000 nm.^9^, ^10^ MTS are prepared following a common sol–gel approach in the presence of a surfactant, where well‐structured mesopores between approximately 3–10 nm are generated.^11^, ^12^, ^13^ Yet, the synthesis of MTS is usually limited to producing powders as other morphologies are especially challenging to obtain. Hence, Galarneau and co‐workers found an alternative way for post‐synthetic micellar induced pore generation in silica materials of different morphology.^14^ This so‐called pseudomorphic transformation (gr. *pseudos*=false, *morphe*=form) process is based on the dissolution of amorphous silica phase in basic media and its recondensation in the presence of a surfactant. Under optimal conditions these competing reactions occur in spatial proximity generating mesoporous network, while the initial morphology of the silica phase can be preserved.^14^, ^15^, ^16^ Enke et al. and Grandjean et al. adapted the process of the pseudomorphic transformation to CPGs with different initial pore diameters ranging from *meso* to macroscales.^17^, ^18^, ^19^ In the case of mesoporous CPGs, the initial pore system collapses during the transformation, but a hierarchically porous silica material can be generated from macroporous CPGs.^20^ The mesopores which are subsequently generated within the CPG pore walls are thus accessible through the macropores, although swelling of the transformed material occurs which reduces the pore diameter of the initial CPG.^20^, ^21^ Furthermore, different concepts for adjustment of the transformation degree have been discussed, for example, by changing the transformation time, the pH value or the ratio of surfactant to silica, intending to preserve the initial pore system.^17^, ^22^ The impact of hierarchical porosity on various applications was recently reviewed.^23^ Moreover, Schwieger et al. gave a good overview on what makes up a hierarchical material and how hierarchical systems can be classified.^24^ Hierarchy does not only mean pores of different sizes but also implies interplay between the different pore levels that improves the flow for example, the distribution of a gas or a liquid.

The incorporation of organic compounds in the silica matrix expands the range of possible applications and makes the synthesis of materials with application‐adjusted features possible. Most common examples are grafting or co‐condensation of silylated organic compounds. Particularly, in the case of small molecules, high organosilica content and good distribution between organic moiety and silica species are feasible by co‐condensation.^25^ In addition, organosilicas that contain up to 80 wt % of organic function and possess various porosity features can be obtained from bridged silsesquisiloxanes (O_1.5_Si‐R‐SiO_1.5_).^26^

The adaption of the micelle templating process leads to periodic mesoporous organosilicas (PMOs), which are a class of materials presenting both molecular scale periodicity and mesoscale ordering of the pores. These organosilica provide very diverse chemical functionality but again limited particle size and shape just as in the case of MTS. Thus, the research has been restricted to mainly nanoparticles.^27^, ^28^ Therefore, broadening the possible range of particle morphology and size as well as chemical functionality in porous organosilica has been an important issue. However, only a few studies have been reported up to now. Reber and Brühwiler added 3‐aminopropyltriethoxysilane (APTES) during the pseudomorphic transformation of silica particles and hence, combined the pseudomorphic transformation with a co‐condensation process.^29^ Our group has utilized the pseudomorphic transformation of organosilica materials with different morphologies, namely of inverse opals and millimeter‐sized organosilica beads.^30^, ^31^

In this work, we combine the morphological flexibility and the macroporosity of CPGs with the chemical functionality of organosilica materials, to produce hierarchically porous organosilica/silica hybrid materials. We chose the precursor 1,4‐((*E*)‐2’‐bis(triethoxysilyl)vinyl)benzene (BTEVB) to incorporate chemical functionality since it has been well established for PMO synthesis for several years.^32^, ^33^, ^34^ Moreover, various functionalized divinylbenzene (DVB)‐bridged analogues, for example, with an amino function^35^ or fluorine,^36^ are also available, which makes the concept extensible for various functionalities. In order to generate hierarchically porous structures, we adapt the concept of pseudomorphic transformation to the impregnated organosilica/silica hybrid material (DVB‐bridged silica@CPG). Using physisorption (suitable for characterizing *meso*‐ and micropores) and mercury intrusion porosimetry (MIP, for characterizing macropores), pore sizes and pore volumes before and after the pseudomorphic transformation have been determined, to prove the formation of hierarchically porous systems. SEM and TEM images have been obtained to follow the changes in morphology and porosity feature. However, it is still not well understood whether the pseudomorphic transformation affects both silica and organosilica species and how the pseudomorphic transformation influences the interconnectivity of the silica and the organosilica phase. The chemical identities of the newly formed mesopore surfaces are not clear as well. Solid state NMR spectroscopy has proven a powerful technique to study chemical/morphological structure and interfacial interaction of various silicate compounds.^37^, ^38^, ^39^, ^40^, ^41^ Thus, we utilized 2‐dimensional (2D) ^29^Si{^1^H} and ^13^C{^1^H} heteronuclear correlation (HETCOR) magic angle spinning (MAS) NMR experiments to investigate the distribution of the organosilica and the silica phases in the hybrid material and the interactions of both Si species with the surfactant during the micelle templating process of the pseudomorphic transformation. Based on the NMR results, structural model for organosilica/silica hybrid material resulting from pseudomorphic transformation will be proposed. The presence of nanopores interconnected with macropores in one particle can provide high flow of fluids and gas, good accessibility to the specific active sites and retention of reactants at the catalytic sites. Therefore, our new approach to form hierarchically porous organosilica/silica hybrid materials can be applied to various fields of technologies from energy storage or catalysis to separation process.^24^

## Results and Discussion

**Figure 1:**
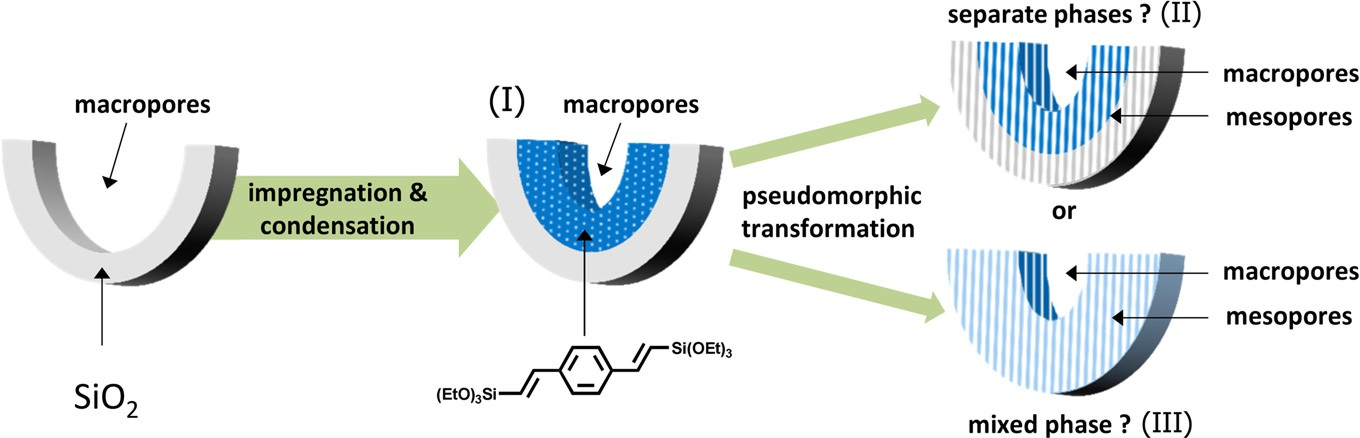
Diagram of the two‐step synthesis (impregnation and subsequent pseudomorphic transformation) of organosilica/silica hybrid materials. After pseudomorphic transformation, either multilayer structure composed of two separate phases of organosilica (blue) and silica (gray) or one mixed phase (light blue) can be formed.

The synthesis process for hierarchically porous organosilica/silicas is subdivided into 1) the impregnation of the CPGs with a solution of the organosilica precursor and its subsequent condensation and 2) the pseudomorphic transformation of the product. We explored this two‐step synthetic protocol since one step process, that is, addition of DVB‐precursor in basic aqueous media to the CPGs in the presence of surfactants did not lead to one hierarchically nanoporous structure but to the formation of a separate organosilica particle. Hence, we found out that a covalent bonding of organosilica to the silica support is prerequisite before the pseudomorphic transformation. Figure 1 gives an overview of the synthetic concept and possible structures. The distribution and interconnection of the organosilica and the silica species in one mixed or two separate phases after the pseudomorphic transformation is one of the key questions and will be investigated intensively by 2D MAS NMR experiments at the end of this work.

### Impregnation of CPG silica with an organosilica phase

**Figure 2:**
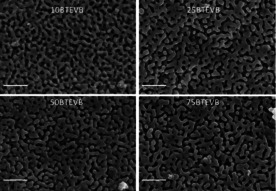
SEM images of impregnated CPGs prepared with 10–75 wt % BTEVB in the impregnation solution (magnification:5×10^4^, scale bar: 1 μm).

In order to combine CPG and DVB‐organosilica, the macroporous system of CPG granules was impregnated with a BTEVB‐containing solution via incipient wetness. The formation of a homogeneous organosilica layer inside the CPG pore system was assumed, where the layer thickness should be adjustable via the organosilica content in the impregnation solution. The BTEVB content in the toluene‐based impregnation solution was varied between 10–75 wt %, and the resulting impregnated materials are denoted as 10BTEVB, 25BTEVB, 50BTEVB and 75BTEVB where the numbers represent the weight percentage (wt %) of BTEVB in the impregnation solution. The SEM images of the impregnated samples in Figure 2 give a first insight about the general trend of pore wall thickness. An SEM image of the initial CPG is given in Figure S1. Due to carbon sputtering of the samples, SEM images are not suitable to estimate the pore size in the materials, however, thickening of the pore walls with increasing organosilica content in the impregnation solutions is clearly visible.

**Figure 3:**
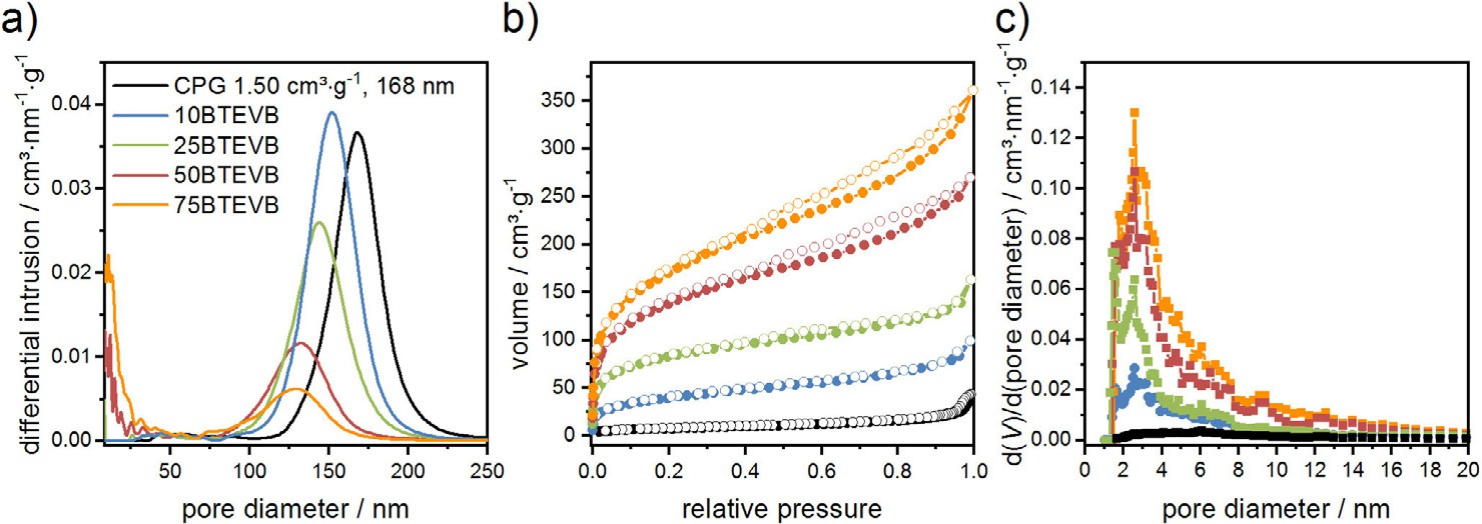
(a) Pore diameter distributions from MIP, (b) N_2_ physisorption isotherms (77 K), and (c) pore diameter distributions from N_2_ physisorption (NLDFT kernel from adsorption branch for silica with cylindrical pores) of CPG and impregnated 10–75BTEVB hybrid materials. MIP and N_2_ physisorption are used to obtain quantitative information about macroporosity and mesoporosity, respectively.

Quantitative information about macroporous media can be obtained from mercury intrusion porosimetry (MIP). The pore diameter distributions and the textural data of the impregnated samples are shown in Figure 3 a and Table 1, respectively (for intrusion curves see the Figure S2). With increasing BTEVB content in the impregnation solution, the macropore diameter maxima as well as the pore volumes decrease. For example, the pore diameter and the pore volume are reduced from 168 nm and 1.5 cm^3^ g^−1^ (initial CPG) to 132 nm and 0.70 cm^3^ g^−1^ for 50 BTEVB. The pore diameter of 75BTEVB is not significantly reduced in comparison to 50BTEVB which indicates that impregnation with higher organosilica content causes blocking of the pores rather than forming a thicker organosilica layer. It appears that the impregnation is less homogeneous with increasing organosilica content in the impregnation solution. With the pore volumes and the weight differences before and after the impregnation, the filling degree could be calculated (see details in the Supporting Information). 75BTEVB has the highest filling degree of 33 % and 25BTEVB and 50BTEVB show the similar filling degree of 21–22 %. In the case of 10BTEVB the pore volume increases slightly after the impregnation which might be due to washing‐out of the finely dispersed silica residues or etching of the silica phase during the harsh alkaline treatment. Nonetheless, shifts of the pore size towards smaller values indicate that divinylbenzene (DVB)‐organosilica is successfully incorporated into the macropores of CPG. The values of the filling degrees suggest that, although incipient wetness was used, 100 % filling is impossible to achieve. This is, on the one hand, due to the change in the density of the organosilica phase after condensation and might, on the other hand, indicate that the organosilica layer is also on the outer surface of the granules.

**Table 1:** Textural data of organosilica/silica hybrid materials prepared by impregnation of CPG.

	S_BET_ [m^2^ g^−1^]^[a]^	*V*_meso_, _NLDFT_ [cm^3^ g^−1^]^[b]^	*d* [nm]^[c]^	*V* [cm^3^ g^−1^]^[c]^	Filling degree [%]^[c]^	TG mass loss [%]^[d]^	Organosilica content [%]^[d]^
CPG	26	0.03	168	1.5	–	–	–
10BTEVB	137	0.11	153	1.57	–	4.6	2
25BTEVB	299	0.19	144	1.14	21	9.4	5
50BTEVB	476	0.35	132	0.69	22	16	11
75BTEVB	596	0.45	130	0.64	33	19	14

[a] Apparent BET specific surface area from N_2_ physisorption (determined considering an adjusted relative pressure range according to the IUPAC recommendation).^44^ [b] Mesopore volumes from N_2_ physisorption (calculated with NLDFT kernel for silica with cylindrical pores from the adsorption branch). [c] Pore diameters (d), volumes (V), and filling degree of macropores from MIP. [d] From TGA.

To characterize mesoporosity, the N_2_ physisorption isotherms of the materials after the impregnation have been acquired as shown in Figure 3 b. The isotherm of the initial CPG is type II as expected for a macroporous material, characterized by unrestricted monolayer‐multilayer adsorption with an almost linear middle section.^43^ The isotherms of 10–75BTEVB shows an additional steep gas uptake in the low relative pressure range which indicates the formation of micro‐ or small mesopores in the impregnated materials (the isotherms are a combination of type Ib and type II). The type Ib characteristics showing gas adsorption in the low‐pressure range are more distinct with increasing organosilica content. Apparent specific BET surface area also increases significantly with increasing organosilica content in the impregnation solution from 137 to 596 m^2^⋅g^−1^ (Table 1). The pore diameter distributions of the hybrid materials 10–75 BTEVB, calculated with a NLDFT kernel, are shown in Figure 3 c. The formation of micropores/small mesopores is notable in all samples, exhibiting increase of mesopore volume upon increasing organosilica content. Here, note that the mesopore volume of newly formed pores is different from the pore volume of initial macroporous system of CPG. We define the mesopore volume (*V*_meso_) as the pore volume originating from the pores smaller than 20 nm to avoid confusion. For condensation of the organosilica phase and its covalent bonding to the silica surface of the CPG, the materials were treated with a solution of ethanol and NaOH at 80 °C. The same treatment of pure BTEVB precursor was found to generate microporous organosilicas with very high surface areas up to 1300 m^2^ g^−1^, whereas the pure CPG is nearly unaffected by such treatment (see physisorption isotherms of both phases after treatment in the Supporting Information Figure S3 and S4). Likely, the additional micropores/small mesopores of the impregnated material results from a nanoporous organosilica layer.

The organosilica content of the impregnated samples can be estimated from the mass loss in TG analysis. As can be seen in the TGA plots in Figure S5, the main mass loss occurs in the temperature range of 300–800 °C, where breakdown of organic moieties and formation of pure silica phase are assumed. The mass losses and the calculated organosilica contents of the samples are given in Table 1. There is a linear relation between the specific BET surface area of the impregnated materials and the DVB‐organosilica content, confirming that the porosity is dominantly influenced by the organosilica content.

The results show that the impregnation of macroporous CPG with BTEVB‐containing solutions and subsequent condensation is an effective method for the synthesis of organosilica/silica hybrid materials. It combines the flexible morphology of the CPG, in this case granulate, with the chemical flexibility of organosilicas. Due to the formation of a nanoporous organosilica layer inside the initial CPG pore system, a hierarchical pore structure is obtained, where the organosilica layer is easily accessible. Hence, this material is of interest in versatile applications for example, for gas adsorption. However, certain limitation still exists due to the very small pore sizes of the nanopore systems.

### Pseudomorphic transformation of impregnated CPG

**Figure 4:**
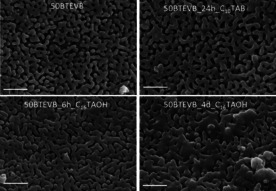
SEM images 50BTEVB after pseudomorphic transformation with C_10_TAB for 24 hours and with C_16_TAOH for 6 hours and 4 days (magnification: 5×10^4^, scale bar: 1 μm).

**Figure 5:**
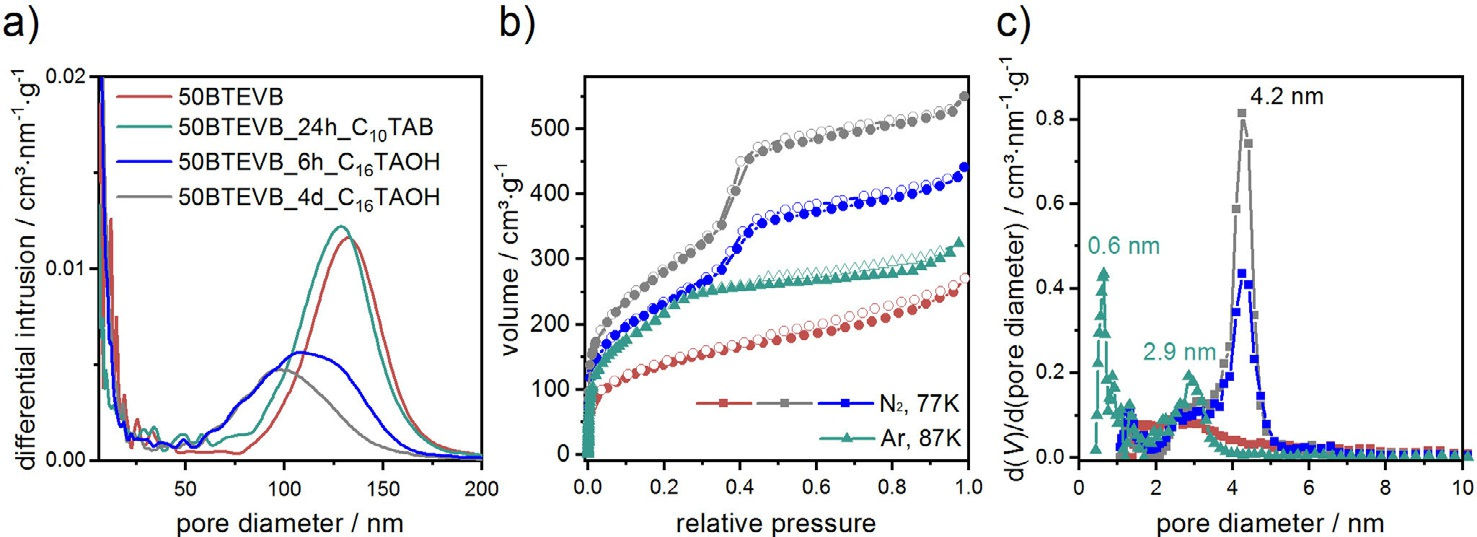
(a) Pore diameter distributions from MIP of organosilica/silica hybrid materials after pseudomorphic transformation. (b) N_2_ and Ar physisorption isotherms (77 K for N_2_ shown in circles, 87 K for Ar shown in triangles) and (c) pore diameter distributions of 50BTEVB before and after pseudomorphic transformation with C_16_TAOH and C_10_TAB surfactants deduced from physisorption (NLDFT kernel from adsorption branch for silica with cylindrical pores in case of N_2_ and for silica/zeolites in case of Ar physisorption). The transformed samples are denoted as 50BTEVB_6 h_C_16_TAOH, 50BTEVB_4 d_C_16_TAOH, and 50BTEVB_24 h_C_10_TAB according to the surfactant and transformation time.

It has been shown that the pseudomorphic transformation can be used to generate second homogeneous mesopores in CPG (pure silica phase).^18^, ^20^, ^21^, ^22^ On the contrary, the effect of pseudomorphic transformation on the organosilica phase and the difference in the reaction kinetics between silica and organosilica phases are not well known. Therefore, we aim to investigate the influence of pseudomorphic transformation on the organosilica phase and to explore the feasibility of improving the porosity features of organosilica/CPG hybrid materials by pseudomorphic transformation. 50BTEVB was chosen exemplarily and two types of surfactants with different aliphatic chain lengths (C_16_TAOH and C_10_TAB) were utilized to demonstrate the tunability of the pore diameter of the second mesopore system. 50BTEVB was treated with C_16_TAOH, which combines the surfactant and the hydroxide ion in one molecule. This enables the synthesis of sodium free materials which are hydrothermally more stable.^17^, ^45^ The transformation time was varied between 4 days (50BTEVB_4 d_C_16_TAOH) and 6 hours (50BTEVB_6 h_C_16_TAOH) to examine the adjustable transformation degree over time.^18^ The pseudomorphic transformation was also carried out with C_10_TAB in NaOH solution for 24 hours (50BTEVB_24 h_C_10_TAB). As shown in SEM images (Figure 4), overall, the morphology of organosilica/CPG hybrid materials is preserved after the pseudomorphic transformation. However, swelling of the material and shrinking of the initial pores are also noted. Significant swelling is observable after four days of reaction time (50BTEVB_4 d_C_16_TAOH), but it is less distinctive with shorter reaction time (50BTEVB_6 h_C_16_TAOH) and negligible in the case of smaller surfactant (50BTEVB_24 h_C_10_TAB). This can be confirmed by the pore diameter distributions from MIP, shown in Figure 5 a (the intrusion curves are given in Figure S2). The pore diameter maxima as well as the pore volumes of macropores decrease significantly after pseudomorphic transformation with C_16_TAOH solution: 50BTEVB_4 d_C_16_TAOH exhibits highest reduction to 100 nm and 0.43 cm^3^ g^−1^, respectively, but for 50BTEVB_6 h_C_16_TAOH with lower transformation degree the macropore diameter decreases less (110 nm). In the case of pseudomorphic transformation with C_10_TAB, the macropore diameter distribution is nearly unchanged compared to the sample before the pseudomorphic transformation (50BTEVB). Hence, the transformation with C_10_TAB causes less swelling of the material which might be due to either the formation of smaller mesopores or incomplete transformation of the hybrid material. The textural data are summarized in Table 2.

**Table 2:** Textural data of organosilica/silica hybrid materials after pseudomorphic transformation.

	S_BET_ [m^2^ g^−1^]	*V*_meso, NLDFT_ [cm^3^ g^−1^]	*D* [nm]	*D* [nm]^[c]^	*V* [cm^3^ g^−1^]^[c]^
50BTEVB	476^[a]^	0.35^[b]^	2–8^[b]^	132	0.69
50BTEVB_6 h_C_16_TAOH	835^[a]^	0.61^[b]^	4.2^[b]^	110	0.52
50BTEVB_4 d_C_16_TAOH	1007^[a]^	0.77^[b]^	4.2^[b]^	100	0.43
50BTEVB_24 h_C_10_TAB	668^[d]^	0.40^[e]^	0.6; 2.9^[e]^	129	0.69

[a] From N_2_ physisorption. [b] Pore volume and pore diameter of nanopores from NLDFT kernel for silica with cylindrical pores (ads. branch of N_2_ physisorption). [c] Pore diameter and pore volume of macorpores from MIP. [d] From Ar physisorption. [e] From NLDFT kernel for silica/zeolites with cylindrical pores (ads. branch of Ar physisorption).

**Figure 6:**
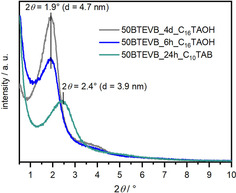
P‐XRD patterns of organosilica/silica hybrid materials after pseudomorphic transformation.

To investigate the change in the mesoporosity caused by pseudomorphic transformation, N_2_ and Ar physisorption measurements are carried out. The N_2_ physisorption data of the samples treated with C_16_‐surfactants show type IVb isotherms with a steep step due to pore condensation at a relative pressure of 0.3–0.5 and no hysteresis (Figure 5 b). The specific BET surface area increases from 476 m^2^ g^−1^ before transformation to 1007 m^2^ g^−1^ and 835 m^2^ g^−1^ after 4 days and 6 hours transformation, respectively (Table 2). In both cases, mesopores of 4.2 nm in diameter are generated, as shown in the pore diameter distributions in Figure 5 c. In addition, the powder X‐ray diffraction patterns (*p*‐XRD, Figure 6) of both materials show one broad reflection at 2*θ*=1.9° (*d=*4.7 nm) which indicates successful templating with the surfactant, although the low resolution of the *p*‐XRD patterns do not allow assignment to a specific phase for example, an MCM‐41‐type structure. This is consistent with the results of pseudomorphic transformed silica phases in the literature.^46^, ^47^ The product treated with the surfactant with shorter aliphatic chain length (50BTEVB_24 h_C_10_TAB) shows *meso*‐ and microporosity and was thus characterized with Ar physisorption (Figure 5 b). The isotherm exhibits high gas uptake at low relative pressure (type Ib) and transits to a steep step due to capillary condensation in small mesopores. Hence, the isotherm is a combination of types Ib and IVb. In comparison to the material before pseudomorphic transformation, increased specific BET surface area of *S*_BET_=668 m^2^ g^−1^ is obtained (following the IUPAC recommendation in the Supporting Information).^44^ Hysteresis occurs which can be due to either interparticle cavitation or desorption from some of the large initial pores through the newly formed mesopores. Similar cavitation effects have also been observed in pseudomorphic transformed CPGs before.^16^, ^17^ The shape of the isotherms indicates the presence of small mesopores and micropores, as can be seen in the pore diameter distribution with two maxima at 0.6 nm and 2.9 nm (Figure 5 c). In addition, *p*‐XRD patterns indicate formation of smaller mesopores when C_10_TAB surfactant is used in comparison to C_16_TAOH (Figure 6). These results are consistent with our expectation, demonstrating that the surfactant with shorter aliphatic chain length generates smaller mesopores and thus, the size of mesopores can be tuned by the choice of the surfactants for pseudomorphic transformation.

**Figure 7:**
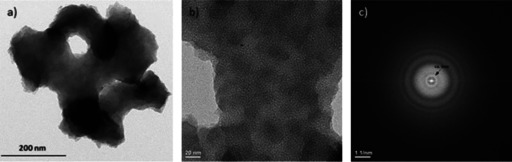
(a), (b) TEM images of 50BTEVB_24 h_C_10_TAB with different magnifications (scale bar given in the image). (c) FFT diffraction pattern of (b).

Additionally, the presence of macro‐ and mesopores in the hybrid material after pseudomorphic transformation is confirmed by TEM, as shown exemplary for 50BTEVB_24 h_C_10_TAB in Figure 7. To obtain TEM images, the material was ground and thus, only fragments of the initial granulates are observed. Figure 7 a shows a fragment with one macropore, exhibiting rough surface and irregular pore shape due to the transformation process. The TEM images indicates full transformation of the complete material and homogeneous distribution of mesoporous network (Figure 7 b). This image was converted into a diffraction pattern by fast Fourier transformation (FFT, Figure 7 c), wherein a periodic distance of around 3 nm can be detected. This is in good agreement with the results for the pore diameter (2.9 nm) and the d‐value from pXRD (3.9 nm) for this material. For TEM images of the initial CPG and 50BTEVB see Figure S6.

### Chemical structure changes by pseudomorphic transformation

**Figure 8:**
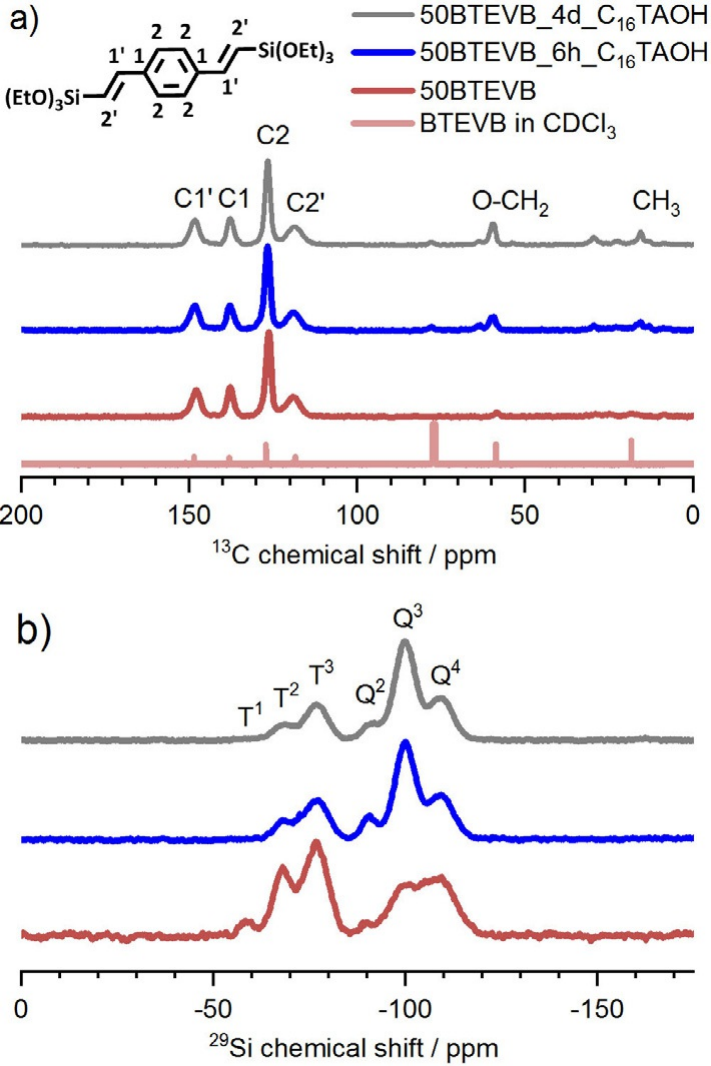
(a) ^13^C CP MAS NMR spectra of 50BTEVB, 50BTEVB_6 h_C_16_TAOH and 50BTEVB_4 d in_C_16_TAOH in comparison to the liquid‐state NMR of BTEVB (CHCl_3_ at 77.16 ppm). (b) ^29^Si CP MAS NMR spectra of these samples. The assignments to the respective C and Si species are shown above the signals.

Chemical structure and the structural stability of organic bridge and siloxane linkage before and after pseudomorphic transformation are examined by ^13^C and ^29^Si CP MAS NMR spectroscopy. Figure 8 a compares the ^13^C CP MAS NMR spectra of 50BTEVB, 50BTEVB_6 h_C_16_TAOH and 50BTEVB_4 d_C_16_TAOH with the liquid‐state NMR of the precursor molecule BTEVB in CDCl_3_. The spectra are in good agreement with each other, indicating the integrity of the organic bridge after the treatments despite the harsh alkaline conditions. Signals between 150–120 ppm result from the divinyl‐benzene bridge. The signals at 17 and 59 ppm are assigned to the ethoxy group bound to partially condensed siloxane species (ex, Q^3^, Q^2^, Q^1^, T^2^, T^1^). Additional weak signals between 10 and 80 ppm are observed for transformed samples, which can be assigned to the aliphatic chain of the surfactant. The intensity of these additional signals is very low, implying that the amount of the remaining surfactant after extraction is not significant.

The ^29^Si CP MAS NMR spectra (Figure 8 b) give semi‐quantitative information about the condensation degree and structural integrity of siloxane network. Contrary to CPG presenting only Q signals of SiO_2_, the impregnated samples exhibit both T (RSi(O)_3_)^48^ and Q (Si(O)_4_)^49^ signals as expected. In pure organosilica materials, the integrity of the Si−C bond can be proven by the absence of Q signals. However, this cannot be applied to the organosilica/silica hybrid materials since Q species is already present due to the silica species of CPG. In the case of 50BTEVB, T^1^ (RSi(OSi)(OR′)_2,_ R′=H or CH_3_CH_2_)^,^ T^2^ (RSi(OSi)_2_(OR′)) and T^3^ (RSi(OSi)_3_) species are observed at −59, −67 and −77 ppm.^32^, ^33^ After the pseudomorphic transformation, the T^1^ signal disappears and relative signal intensity of T^3^ over T^2^ species increases, which indicates increase in the condensation degree and formation of highly networked organosilica structure. It appears that the mechanical stability of the organosilica phase can benefit from the transformation process. Additionally, significant change is seen in Q species. Before the pseudomorphic transformation, the ratio of Q^3^ (Si(OSi)_3_(OR′)) to Q^4^ (Si(OSi)_4_) signals is nearly 1:1, whereas after transformation, the intensity of Q^3^ signal increases significantly and becomes dominant. This behavior can also be observed for pure CPG after pseudomorphic transformation, implying that the silica phase is affected by the transformation process as well (Figure S8). The spectra of 50BTEVB_6 h_C_16_TAOH and 50BTEVB_4 d_C_16_TAOH are very similar.

In the impregnated samples, the mass loss in TG was used to evaluate the organosilica content in the hybrid materials, but after the transformation, this method turned out to be insufficiently reliable (TG/MS data in Figure S9): the mass loss in the considered temperature range is higher (20–21 %) than before the transformation (16 %), which might be due to the presence of remaining surfactant and increase in ethoxy group as suggested in ^13^C and ^29^Si MAS NMR. Instead, ^29^Si MAS NMR with direct excitation can be used for reliable quantification of the different silica species. Note that CP MAS NMR results do not provide quantitative information because signal intensity depends on the functional group and mobility of the molecule. Since direct excitation method is time consuming, only 50BTEVB and 50BTEVB_6 h_C_16_TAOH were measured. The spectral deconvolution of signals and the quantitative results can be found in Figure S10 and Table S3. DVB‐organosilica content in the hybrid material can be deduced from the intensity ratio of T/Q signals, showing decrease of organosilica content from 16 to 8.1 mol % after the transformation. This reduction can be due to leaching of the organosilica phase during the transformation or cleavage of the Si−C bond. Partial cleavage of the Si−C bond can occur, leading to higher ratios of Q signals and lower ratios of T signals at the same time. Other examples in the literature also suggest that the small fraction of Si−C bond undergoes cleavage and formation into Q species after pseudomorphic transformation of organosilica materials.^30^, ^31^

Overall, 1D NMR results confirm that impregnation of CPG leads to the formation of DVB‐bridged organosilica phase inside the CPG macropores and after pseudomorphic transformation, DVB‐bridged organosilica phase remains incorporated in the organosilica@CPG hybrid materials. Changes in the relative ratio among T groups suggest that mesopores are formed in organosilica phase by pseudomorphic transformation. However, we cannot rule out the possibility that the mesoporous structures are also generated in silica phase. The exact structural changes are investigated by multi‐dimensional NMR spectroscopy as below.

### Phase transition by pseudomorphic transformation

Now, a question arises: how are each T and Q species and pore systems distributed in the hybrid material after the pseudomorphic transformation? The hybrid material can either form multi‐layer structure consisting of separate organosilica and silica phases or a single layer structure composed of random distribution of both T and Q silicon species. To answer these questions, 2D frequency‐switched Lee‐Goldburg heteronuclear correlation (FSLG‐HETCOR) MAS NMR spectroscopy is utilized since HETCOR is a powerful technique to detect correlation of nuclear spin pairs which reside in close proximity in space, thus providing information about the molecular assembly structure and geometrical arrangements.^50^, ^51^, ^52^

**Figure 9:**
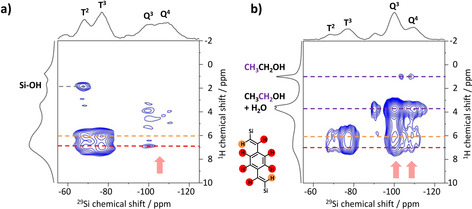
^29^Si{^1^H} FSLG HETCOR MAS NMR spectra of (a) 50BTEVB and (b) 50BTEVB_6 h_C_16_TAOH acquired with 2 ms contact time. Corresponding 1D ^1^H MAS and ^29^Si CP MAS NMR spectra are shown along the vertical and horizontal axis, respectively. The spectra are plotted with contour levels from 20 % of the intensity of the DVB‐group signal. The assignments of the correlation signals to the corresponding protons are denoted by lines with a color code (red, orange: DVB group, purple: ethoxy, grey: hydroxy group).

First, the impregnated CPG material (50BTEVB) was characterized by 2D ^29^Si{^1^H} FSLG‐HETCOR NMR measurements as shown in Figure 9 a. As expected, intramolecular correlation signals belonging to DVB‐bridged silica species are predominantly observed: signals of T^2^ (^29^Si chemical shift at −68 ppm) and T^3^ (^29^Si at *δ*=−77 ppm) species are correlated with ^1^H signals of divinylbenzene (DVB) at *δ*=6 and 7 ppm, as indicated by orange and red lines in the Figure, respectively. The signal at 6 ppm corresponds to the vinyl protons which are closer to silicon and the correlation at 7 ppm can be assigned to the second vinyl proton and the aromatic protons. The second vinyl protons and the aromatic protons cannot be distinguished clearly since the difference in NMR frequency of these moieties is smaller than the linewidth of solid‐state NMR (e.g. 7.19 and 7.45 ppm for 2^nd^ vinyl and aromatic protons, respectively, of the precursor BTEVB in CDCl_3_). The correlation signal between Q Si species and the protons of the organic bridge are of very low intensity (marked by arrow). This indicates that only small amounts of the organosilica bridge are in contact with pure silica, for example, at the phase boundary, but the two species are not mixed. This confirms our expectations for the formation of two‐layer structure consisting of two independent silica and organosilica phases (structure Ι in Figure 1). Correlation between isolated silanol groups and T^2^ Si occur between ^29^Si at *δ*=−68 ppm and ^1^H at *δ*=1.8 ppm (marked by a grey line), which is a common observation for zeolitic materials.^38^, ^40^, ^53^, ^54^

Figure 9 b shows ^29^Si{^1^H} FSLG‐HETCOR NMR of 50BTEVB_6 h_C_16_TAOH. After pseudomorphic transformation, DVB‐bridge group (^1^H, at *δ*=6–7 ppm) yields additional strong correlation peaks with Q^3^ (^29^Si at −110 ppm) and Q^4^ (−110 ppm) Si species (marked by arrow). This strong correlation between DVB‐bridge and Q species indicates that T and Q species are now in close contact with each other, suggesting that the material does not form two separate layers, but these two species are randomly mixed at the molecular level. During the pseudomorphic transformation, Q Si species from the inner part of the particles can be dissolved and progressively recondensate with T Si species at the pore surfaces, which is suggested by the swelling of the pore wall structure as seen in the SEM images in Figure 4. In addition, cleavage of the Si−C bond and recondensation of the resulting Q species with uncleaved T species possibly occur, yielding the mixture of T and Q species. Additional cross peaks at δ(^1^H)=3.8 ppm and 0.9 ppm are caused by correlation of the Q species with ethoxy group, ethanol and water.

### Micelle templating of organosilica and silica species

The formation of a second mesopore system shown by N_2_ physisorption, TEM and *p*‐XRD measurements demonstrates successful pseudomorphic transformation of the organosilica/silica hybrid material. However, among the organosilica and the silica species, it is not clear which species are affected by the micelle templating process and of which species the pore walls are composed. To answer this question, a pseudomorphic transformed sample before the surfactant extraction has been prepared and the interfacial structure between the silica species and the surfactant has been investigated. The signals of the surfactants are dominantly seen in ^1^H and ^13^C CP MAS NMR spectra, confirming the presence of the surfactant (NMR spectra with the peak assignments are shown in Figure S11). In particular, ^13^C NMR signal of inner methylene chain (‐CH_2_‐) and chain‐end methyl group appear at 30.3 and 14.2 ppm, respectively, implying that the aliphatic chains of the surfactants assume gauche‐rich conformation in contrast to the *trans* conformation of bulk‐phase. This is consistent with the gel‐like behavior of the surfactants confined in nanopores.^38^, ^55^ No additional signals that can be assigned to the surfactants in the bulk phase are seen, indicating that the surfactants are located predominantly within the pores.

**Figure 10:**
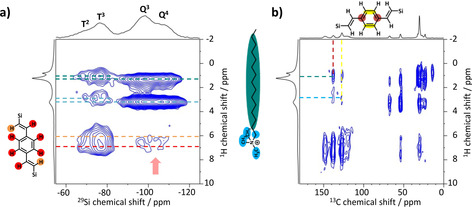
2D NMR spectra of 50BTEVB_incl. 6hC_16_TAOH. (a) ^29^Si{^1^H} FSLG HETCOR MAS NMR spectrum (contact time=5 ms, contour levels from 20 % of the signal intensity of DVB‐T^3^ correlation) and (b) ^13^C{^1^H} FSLG HETCOR MAS NMR spectrum (contact time=4 ms, contour levels from 10 % of the signal intensity of DVB intramolecular correlation). The assignments of the correlation signals to the corresponding protons are denoted by lines with a color code (red, orange: DVB group, light blue: surfactant head group, green: surfactant inner ‐CH_2_‐ chain).

Spatial proximity between the surfactants and each Si groups are investigated by the 2D ^29^Si{^1^H} FSLG HETCOR MAS NMR of 50BTEVB_6 h before extraction (incl.C_16_TAOH) as shown in Figure 10 a. Again, correlations with DVB group (^1^H NMR, *δ*=6–7 ppm) are seen for Q species (marked by arrow), confirming mixed phase at the molecular level instead of separate domain structures. In addition, strong correlation of Q^4^ and Q^3^ Si species are observed with ^1^H at 3.2 and 1.3 ppm, which can be assigned to the methyl/ethyl group (CH_3_N/NCH_2_‐) of the surfactant's head group and inner methylene chain, respectively (denoted by blue and green lines in Figure). The correlation between Q group and surfactant indicates that silica species build pore walls and interact with the surfactant inside the pore. In particular, the correlation with the head group is very intense in comparison to that with the inner methylene chain, which is in agreement with the suggested micelle structure: polar head groups of the surfactant are directly interacting with the silica pore wall due to the electrostatic interaction and the long hydrocarbon chains face toward the center of the pores.^11^, ^38^ By contrast, weaker correlation signals of T^3^ and T^2^ groups appear at slightly different chemical shift positions (^1^H, *δ*=2.8 and 1.0 ppm) from those of the correlation with Q species. These correlation peaks cannot be attributed to SiOH, SiOCH_2_CH_3_ and residual water, since silanol and water exhibit correlation mainly with T^2^ and T^1^ species as is seen in Figure 9 a. Thus, we assign this correlation to the interaction between T species and the surfactant. Since NMR is sensitive to the local environments, the surfactants nearby T functional group and Q group can yield signals at different frequencies due to the different chemical environments of the hydrophobic DVB group in comparison to the hydrophilic Q silica species.

A close spatial proximity between the surfactant and T groups are further verified by the ^13^C{^1^H} FSLG HETCOR MAS NMR as show in in Figure 10 b. As expected, intramolecular correlations between aromatic carbons (^13^C, *δ*=120–150 ppm) and aromatic protons (^1^H, *δ*=6–7 ppm) belonging to the DVB‐bridge group and those between aliphatic carbons (^13^C, *δ*=10–70 ppm) and protons (^1^H, *δ*=1–4 ppm) belonging to the surfactants are observed. More interestingly, intermolecular correlation signals between DVB and the head group as well as the inner methylene chain of surfactants are visible as represented by the colored lines in the Figure 10 b. ^1^H chemical shift values of these intermolecular correlation signals are in agreement with those between T Si species and surfactants observed in ^29^Si{^1^H} FSLG HETCOR (Figure 10 a), which confirms our assignment of surfactants interacting with T and organic bridge groups. The correlation of the surfactant with T species as well as with the DVB group indicates that organosilica species and the surfactants are in close contact with each other and thus, pore walls also consist of organosilica species.

Overall, 2D NMR results clearly confirm that both silica and the organosilica species are affected by the micelle templating, resulting in the formation of mesopores which are composed of both T and Q Si species. Further, silica and organosilica species co‐condense during the pseudomorphic transformation, yielding a single mesophase of which pore walls consist of a random mixture of T and Q species at the molecular level (structure III in Figure 1). Thus, a new type of single‐phase mesoporous morphology is obtained by pseudomorphic transformation from two‐layer system of separate silica and organosilica phases. Two‐layer structure could suffer from peeling‐off and fragmentation of organosilica layer from the silica framework which would deteriorate the performance in actual applications. Likely, single‐phase structure is beneficial since the mixing and co‐condensation of organosilica and silica species could enhance the mechanical strength of the functional organosilica phase and stabilize hierarchically porous structures.

## Conclusions

Hierarchically porous organosilica/silica hybrid materials have been successfully synthesized in a two‐step approach by impregnation and pseudomorphic transformation. First, incipient wetness impregnation was found as a suitable method to form a layer of a nanoporous divinylbenzene‐bridged organosilica phase in the macropores of controlled pore glasses, while maintaining the morphology of CPG. The product exhibits high specific BET surface area of up to 600 m^2^ g^−1^ resulting from micro‐ or small mesopores of organosilica layer. It was shown that the macropore size and the apparent specific BET surface area are adjustable by the organosilica content. Second, the concept of pseudomorphic transformation, which is well known for pure CPGs, was adapted to form hybrid materials. After pseudomorphic transformation of impregnated materials, the hybrid materials show significant increase in the specific BET surface areas up to 1000 m^2^ g^−1^ and an ordered second mesopore system of which pore size and surface area are tunable with the choice of the cationic surfactant. Mesopores of *d=*4.2 nm and 2.9 nm are generated with C_16_TAOH and C_10_TAB surfactants, respectively. The 3‐dimensional shape of CPG remains the same, however, the macropore size shrinks slightly which can be controlled by the transformation parameters. Homogeneous distribution of mesopores throughout the hybrid material is demonstrated by TEM images. Two‐dimensional ^29^Si{^1^H} FSLG HETCOR NMR spectroscopy provides direct evidence for different phase behavior of these hybrid materials by probing correlation between organosilica and silica group. Double layer structure composed of separate organosilica and silica layers (structure I) is formed for impregnated material, while a single mesophase where both T and Q Si species are randomly distributed in the framework is identified for pseudomorphic transformed material (structure III). ^29^Si{^1^H} and ^13^C{^1^H} FSLG HETCOR NMR of the pseudomorphic transformed hybrid material before the surfactant extraction shows correlation signals of surfactant with both T and Q species, indicating that the micellar templating affects both organosilica and silica phases and leads to structuring of pore walls via co‐condensation of T and Q species at the molecular level. This demonstrates that the pseudomorphically transformed materials provide the accessibility to the organic functionality, which is of crucial importance for its potential application, for example, in gas separation processes and catalytic reaction. Additionally, the co‐condensation structure can have a reinforcing effect on the organosilica phase. Our synthetic protocol could be expanded to various bis‐silylated organosilica precursors as well as different porous glasses and hence, it opens a new field of variable hybrid materials with hierarchical porosity and chemical functionality.

## Experimental Section

**Materials**: All compounds were used as received from commercial suppliers: Ampersep®900 hydroxide form (*Sigma Aldrich*), cetyltrimethylammonium bromide (C_16_TAB, *Alfa Aesar*, 98 %), decyltrimethylammonium bromide (C_10_TAB, >99 %, *TCI Chemicals*), ethanol (99.8 %), toluene (99.7 %).

**CPG synthesis**: A sodium borosilicate glass with a constitution of 62 wt % SiO_2_, 30 wt % B_2_O_3_, 6 wt % Na_2_O and 1 wt % Al_2_O_3_ was milled and fractionated according to the grain sizes by sieve. The fraction with a grain class of 40–100 μm was annealed at 620 °C for 24 hours in a muffle furnace for phase separation. For acidic leaching the glass was treated in HCl (3 mol L^−1^) at 90 °C for 6 hours, and then washed until the pH of the washing solution became neutral and dried. For alkaline extraction the glass was stirred in NaOH solution (0.5 mol L^−1^) at 30 °C for 5 hours (volume ratio of solution/glass=1/8). Subsequently, it was treated in NaOH solution (3 mol L^−1^) under static condition at reduced pressure (0.2 bar) and at 0 °C for two hours (volume ratio solution/glass=1:2). Again, the glass was washed until neutral and dried at 120 °C.

**Impregnation of CPG**: Before impregnation the respective CPGs were heated up to 200 °C for at least 4 hours and cooled down to room temperature. 1,4‐((*E*)‐2’‐Bis(triethoxysilyl)vinyl)benzene (BTEVB) was synthesized according to the literature^33^ (detail in ESI). In a typical mixture of the impregnation solution, 100 mg of the BTEVB was mixed with 900 mg of toluene in order to obtain a 10/90 (w/w) mixture under stirring for 10 minutes. For 25/75, 50/50 or 75/25 (w/w) mixtures the masses were adjusted, respectively, and the resulting samples are denoted as 10BTEVB, 25BTEVB, 50BTEVB and 75BTEVB. From this solution 1.5 mL were added dropwise to 1.00 g CPG in a round bottom flask. Impregnation was carried out in a rotary evaporator (*Büchi*) using a defined pressure protocol under rotation: for 5 minutes at 300 mbar, 25 minutes at 100 mbar, 15 minutes at full evaporation power (approx. 9 mbar) at room temperature and another 15 minutes at full power in a water bath at 50 °C. After impregnation, the composite of CPG and the precursor was placed in a screw cap bottle. A solution of 7.5 mL ethanol (99.8 %) and 2.5 mL NaOH solution (0.31 mol L^−1^, pH 13.5) was prepared. 1.5 mL of this solution were added to the composite before it was treated in a sealed screw cap bottle at 80 °C for 20 hours. Afterwards the obtained material was filtered, washed with water and ethanol and finally dried at 80 °C.

**Pseudomorphic transformation of organosilica/silica hybrid materials**: Cetyltrimethylammonium hydroxide **(**C_16_TAOH) solution was prepared in NaOH medium according to the literature.^17^ 500 mg of the organosilica/silica hybrid materials from impregnation were treated in 20 mL C_16_TAOH solution (0.08 mol L^−1^) at 100 °C for 6 hours (_6 h_C_16_TAOH) or 4 days (_4 d_C_16_TAOH) under static conditions. Alternatively, 910 mg C_10_TAB (3.2 mmol) in 24 mL NaOH solution (0.1 mol L^−1^) was stirred at room temperature for 30 minutes before 600 mg of the organosilica/silica hybrid materials were added and the mixture was treated at 100 °C for 24 hours under static conditions (_24 h_C_10_TAB). All materials were filtered, washed with water and ethanol and dried at 80 °C. For extraction of the surfactant the samples were treated in ethanol/hydrochloric acid (32 %) mixture (v/v=97/3) under reflux conditions for three days, and subsequently the materials were filtered, washed with water and ethanol and dried at 80 °C again.

### Characterization

**Physisorption**: All samples were outgassed on a *Quantachrome Degasser Masterprep* under vacuum at 80 °C for 16 hours. N_2_ physisorption data were recorded at 77 K with a *Quantachrome Quadrasorb‐SI‐MP*/ *Quadrasorb evo* or *Quantachrome Autosorb 6B*. Ar physisorptions were recorded at 78 K with *Quantachrome Autosorb®‐iQ‐MP* using *Quantachrome Cryocooler*. The specific surface areas were determined using the method by Brunauer, Emmett and Teller (BET).^42^ The relative pressure range which is considered for determination of the specific BET surface area was adjusted according to the IUPAC recommendation so that it is referred to as the apparent specific BET surface area. Pore diameter distribution was calculated using the non‐local density functional theory (NLDFT) kernel for silica with cylindrical pores from the adsorption branch of N_2_ physisorption, and using NLDFT kernel for silica/zeolites with cylindrical pores from the adsorption branch of Ar physisorption. The mesopore volume was calculated using the same NLDFT kernel, considering pores smaller than 20 nm.

**Mercury intrusion porosimetry (MIP)**: Measurements were performed with a *micromeritics AUTOPORE V SERIES* instrument or *Quantachrome Poremaster*.

**Powder X‐ray Diffraction (*p*‐XRD)**: Diffraction patterns were recorded at room temperature on a *Panalytical MPD X′Pert Pro* by applying filtered Cu‐Kα radiation.

**Thermal analysis (TG/MS)**: *Netzsch STA 449 F3 Jupiter* coupled with mass spectrometer *QMS 403C Aëolos Quadrupol* was used in Ar/O_2_ (80/20) with a flow of 40 mL min^−1^ and heating rate of 5 K min^−1^.

**Scanning electron microscope (SEM)**: *Zeiss Leo 1525 Gemini* was utilized with acceleration voltage of 5 kV using an *Inlense detector* after carbon sputtering of the sample.

**Transmission electron microscope (TEM)**: TEM images were collected utilizing JEOL JEM‐1011 (100 kV) and JEOL JEM 2200 FS (200 kV). The samples were ground using a porcelain mortar and pestle and dispersed in ethanol. The solution was transferred on a standard carbon coated cupper grid (400 mesh) for measurement.

**Solid‐state nuclear magnetic resonance (NMR) spectroscopy**: Solid‐state NMR experiments were performed on Bruker Avance II 400 spectrometer equipped with a 4 mm double resonance magic angle spinning (MAS) probe. ^1^H MAS NMR spectra were acquired with an operating frequency of 400.28 MHz, MAS frequency of 13 kHz, 90° pulse length of 4.2 μs and repetition delay of 3 s. ^13^C cross‐polarization (CP) MAS spectra were collected with ^13^C operating frequency of 100.66 MHz, MAS frequency of 13 kHz, contact time of 1 ms, repetition delay of 4 s, ramped polarization transfer and two‐pulse phase‐modulated (TPPM) proton decoupling. ^29^Si CP MAS NMR spectra were acquired with ^29^Si frequency of 79.52 MHz, contact time of 2 ms, MAS frequency of 5 kHz, recycle delay of 5 s and continuous wave proton decoupling. 2D ^13^C{^1^H} and ^29^Si{^1^H} frequency‐switched Lee–Goldburg (FSLG) heteronuclear correlation (HETCOR) MAS NMR spectra were obtained using a ramped polarization transfer with ^13^C rf field strength of 60 kHz, FSLG homonuclear decoupling during the proton spin evolution time and TPPM heteronuclear ^1^H decoupling during the acquisition. ^1^H rf field strength of 66 kHz were used for both homonuclear and heteronuclear decoupling. Contact times were varied between 2 and 5 ms. 200 increments were recorded in t1 with States‐TPPI method for phase‐sensitive detection. MAS frequency of 13 kHz and repetition delay of 3 s were used.

## Conflict of interest

The authors declare no conflict of interest.

## Supporting information

As a service to our authors and readers, this journal provides supporting information supplied by the authors. Such materials are peer reviewed and may be re‐organized for online delivery, but are not copy‐edited or typeset. Technical support issues arising from supporting information (other than missing files) should be addressed to the authors.

Supplementary

## References

[1] E. Serrano , N. Linares , J. Garcia-martinez , J. R. Berenguer , ChemCatChem 2013, 5, 844–860.

[2] D. Enke , R. Gläser , U. Tallarek , Chem. Ing. Technik 2016, 88, 1561–1585.

[3] W. Haller , Nature 1965, 206, 693–696.

[4] I. Mutavdžin , T. Munkelt , D. Enke , A. Seidel-Morgenstern , Chem. Eng. Technol. 2019, 42, 241–251.

[5] R. Schmidt , M. Stöcker , E. Hansen , D. Akporiaye , O. H. Ellestad , Microporous Mater. 1995, 3, 443–448.

[6] Z. Wu , D. Zhao , Chem. Commun. 2011, 47, 3332–3338. 10.1039/c0cc04909c 21253630

[7] D. I. Fried , F. J. Brieler , M. Fröba , ChemCatChem 2013, 5, 862–884.

[8] K. Engelmark Cassimjee , M. Kadow , Y. Wikmark , M. S. Humble , M. L. Rothstein , D. M. Rothstein , Chem. Commun. 2014, 50, 9134–9137. 10.1039/c4cc02605e 24989793

[9] M. E. Nordberg , J. Am. Ceram. Soc. 1944, 27, 299–305.

[10] A. Inayat , B. Reinhardt , H. Uhlig , W.-D. Einicke , D. Enke , Chem. Soc. Rev. 2013, 42, 3753–3764. 23081802 10.1039/c2cs35304k

[11] J. S. Beck , J. C. Vartuli , W. J. Roth , M. E. Leonowicz , C. T. Kresge , K. D. Schmitt , C. T. W. Chu , D. H. Olson , E. W. Sheppard , S. B. McCullen , J. B. Higgins , J. L. Schlenker , J. Am. Chem. Soc. 1992, 114, 10834–10843.

[12] C. T. Kresge , M. E. Leonowicz , W. J. Roth , J. C. Vartuli , J. S. Beck , Nature 1992, 359, 710–712.

[13] Q. Huo , D. I. Margolese , U. Ciesla , P. Feng , T. E. Gier , P. Sieger , R. Leon , P. M. Petroff , F. Schüth , G. D. Stucky , Nature 1994, 368, 317–321.

[14] T. Martin , A. Galarneau , F. Di Renzo , F. Fajula , D. Plee , Angew. Chem. Int. Ed. 2002, 41, 2590–2592; 10.1002/1521-3773(20020715)41:14<2590::AID-ANIE2590>3.0.CO;2-3 12203544

[15] A. Galarneau , J. Iapichella , K. Bonhomme , F. De Renzo , P. Kooyman , O. Terasaki , F. Fajula , Adv. Funct. Mater. 2006, 16, 1657–1667.

[16] W. D. Einicke , D. Enke , M. Dvoyashkin , R. Valiullin , R. Gläser , Materials 2013, 6, 3688–3709. 28788300 10.3390/ma6093688 PMC5452651

[17] H. Uhlig , M. L. Gimpel , A. Inayat , R. Gläser , W. Schwieger , W. D. Einicke , D. Enke , Microporous Mesoporous Mater. 2013, 182, 136–146.

[18] M. Guillot , S. El Mourabit , J. Ravaux , A. Tokarev , F. Goettmann , A. Grandjean , Microporous Mesoporous Mater. 2014, 197, 83–91.

[19] M. Guillot , F. Goettmann , C. Delchet , S. El Mourabit , A. Grandjean , WO/2014/083162, 2014.

[20] H. Uhlig , J. Hollenbach , M. Rogaczewski , J. Matysik , F. J. Brieler , M. Fröba , D. Enke , Chem. Ing. Tech. 2017, 89, 863–875.

[21] A. Inayat , B. Reinhardt , J. Herwig , C. Küster , H. Uhlig , S. Krenkel , E. Raedlein , D. Enke , New J. Chem. 2016, 40, 4095–4114.

[22] W. D. Einicke , H. Uhlig , D. Enke , R. Gläser , C. Reichenbach , S. G. Ebbinghaus , Colloids Surf. A 2013, 437, 108–112.

[23] M.-H. Sun , S.-Z. Huang , L.-H. Chen , Y. Li , X.-Y. Yang , Z.-Y. Yuan , B.-L. Su , Chem. Soc. Rev. 2016, 45, 3479–3563. 27255561 10.1039/c6cs00135a

[24] W. Schwieger , A. G. Machoke , T. Weissenberger , A. Inayat , T. Selvam , M. Klumpp , A. Inayat , Chem. Soc. Rev. 2016, 45, 3353–3376. 26477329 10.1039/c5cs00599j

[25] F. Hoffmann , M. Cornelius , J. Morell , M. Fröba , Angew. Chem. Int. Ed. 2006, 45, 3216–3251; 10.1002/anie.200503075 16676373

[26] J. G. Croissant , X. Cattoën , J.-O. Durand , M. Wong Chi Man , N. M. Khashab , Nanoscale 2016, 8, 19945–19972. 27897295 10.1039/c6nr06862f

[27] E. De Canck , I. Ascoop , A. Sayari , P. Van Der Voort , Phys. Chem. Chem. Phys. 2013, 15, 9792–9799. 23666594 10.1039/c3cp50393c

[28] J. G. Croissant , Y. Fatieiev , H. Omar , D. H. Anjum , A. Gurinov , J. Lu , F. Tamanoi , J. I. Zink , N. M. Khashab , Chem. Eur. J. 2016, 22, 9607–9615. 27245497 10.1002/chem.201600587

[29] M. J. Reber , D. Brühwiler , Part. Part. Syst. Charact. 2015, 32, 243–250.

[30] T. Simon , F. J. Brieler , M. Fröba , J. Mater. Chem. C 2017, 5, 5263–5268.

[31] M. Bilo , Y. J. Lee , M. Fröba , Microporous Mesoporous Mater. 2019, 284, 327–335.

[32] A. Sayari , W. Wang , J. Am. Chem. Soc. 2005, 127, 12194–12195. 16131176 10.1021/ja054103z

[33] M. Cornelius , F. Hoffmann , M. Fröba , Chem. Mater. 2005, 17, 6674–6678.

[34] C. S. Vogelsberg , S. Bracco , M. Beretta , A. Comotti , P. Sozzani , M. A. Garcia-Garibay , J. Phys. Chem. B 2012, 116, 1623–1632. 22220838 10.1021/jp2119263

[35] M. Beretta , J. Morell , P. Sozzani , M. Fröba , Chem. Commun. 2010, 46, 2495–2497. 10.1039/b923485c 20379561

[36] S. Bracco , M. Beretta , A. Cattaneo , A. Comotti , A. Falqui , K. Zhao , C. Rogers , P. Sozzani , Angew. Chem. Int. Ed. 2015, 54, 4773–4777; 10.1002/anie.201412412 25689486

[37] N. Baccile , G. Laurent , C. Bonhomme , P. Innocenzi , F. Babonneau , Chem. Mater. 2007, 19, 1343–1354.

[38] A. Comotti , S. Bracco , P. Valsesia , L. Ferretti , P. Sozzani , J. Am. Chem. Soc. 2007, 129, 8566–8576. 17579407 10.1021/ja071348y

[39] J. T. A. Jones , C. D. Wood , C. Dickinson , Y. Z. Khimyak , Chem. Mater. 2008, 20, 3385–3397.

[40] Y. Pan , H. Wu , C.-C. Kao , H. Kao , Y. Shieh , G. T. K. Fey , J.-H. Chang , H.-H. G. Tsai , J. Phys. Chem. C 2009, 113, 18251–18258.

[41] J. B. Mietner , F. J. Brieler , Y. J. Lee , M. Fröba , Angew. Chem. Int. Ed. 2017, 56, 12348–12351; 10.1002/anie.201705707 28715619

[42] S. Brunauer , P. H. Emmett , E. Teller , J. Am. Chem. Soc. 1938, 60, 309–319.

[43] M. Thommes , K. Kaneko , A. V. Neimark , J. P. Olivier , F. Rodriguez-Reinoso , J. Rouquerol , K. S. W. Sing , Pure Appl. Chem. 2015, 87, 1051–1069.

[44] M. Thommes , K. Kaneko , A. V. Neimark , J. P. Olivier , F. Rodriguez-Reinoso , J. Rouquerol , K. S. W. Sing , Pure Appl. Chem. 1985, 57, 603–619.

[45] T. R. Pauly , V. Petkov , Y. Liu , S. J. L. Billinge , T. J. Pinnavaia , J. Am. Chem. Soc. 2002, 124, 97–103. 11772066 10.1021/ja0118183

[46] J. Babin , J. Iapichella , B. Lefèvre , C. Biolley , J.-P. Bellat , F. Fajula , A. Galarneau , New J. Chem. 2007, 31, 1907.

[47] H. Uhlig , T. Muenster , G. Kloess , S. G. Ebbinghaus , W. D. Einicke , R. Gläser , D. Enke , Microporous Mesoporous Mater. 2018, 257, 185–192.

[48] M. Geppi , S. Borsacchi , G. Mollica , C. A. Veracini , Appl. Spectrosc. Rev. 2008, 44, 1–89.

[49] M. Magi , E. Lippmaa , A. Samoson , G. Engelhardt , A. R. Grimmer , J. Phys. Chem. 1984, 88, 1518–1522.

[50] D. C. Apperley , R. K. Harris , P. Hodgkinson , Solid-State NMR: Basic Principles and Practice, Momentum Press, New York 2012.

[51] B.-J. van Rossum , C. P. de Groot , V. Ladizhansky , S. Vega , H. J. M. de Groot , J. Am. Chem. Soc. 2000, 122, 3465–3472.

[52] B.-J. van Rossum , H. Förster , H. J. M. de Groot , J. Magn. Reson. 1997, 124, 516–519.

[53] J. Trébosc , J. W. Wiench , S. Huh , V. S.-Y. Lin , M. Pruski , J. Am. Chem. Soc. 2005, 127, 3057–3068. 15740145 10.1021/ja043567e

[54] J.-B. d′espinose de la Caillerie , M. R. Aimeur , Y. E. Kortobi , A. P. Legrand , J. Colloid Interface Sci. 1997, 194, 434–439. 9398426 10.1006/jcis.1997.5126

[55] R. Simonutti , A. Comotti , S. Bracco , P. Sozzani , Chem. Mater. 2001, 13, 771–777.

