# Supplementary material for: Structural Changes of Hierarchically Nanoporous Organosilica/Silica Hybrid Materials by Pseudomorphic Transformation
Source: Chemistry. 2020 Jun 11;26(49):11220–30. doi: 10.1002/chem.202000512 (PMC7497150; doi:10.1002/chem.202000512)
Supplement: Supplementary file 1 — Supplementary [file CHEM-26-11220-s001.pdf]

Chemistry–A European Journal

Supporting Information

Structural Changes of Hierarchically Nanoporous Organosilica/ Silica Hybrid Materials by Pseudomorphic Transformation

Malina Bilo,[a] Maximilian Münzner,[b] Christian Küster,[b] Dirk Enke,[b] Young Joo Lee,*[a] and Michael Fröba*[a]

1. Synthesis of 1,4-((E)-2′-Bis(triethoxysilyl)vinyl)benzene (BTEVB)

2. SEM images of initial CPG batch

3. MIP data and calculation of the filling degree

4. Treatment of CPG or BTEVB with ethanol/ sodium hydroxide solution

5. TGA of organosilica/silica hybrid materials 10–75BTEVB

6. TEM images of the initial CPG and 50BTEVB

7. Specific surface area from combined isotherms of type Ib and IVb isotherm

8. Solid-state NMR spectra of pseudomorphic transformed materials

9. TG/MS data after pseudomorphic transformation

10. Spectral deconvolution of ^{29}Si MAS NMR of pseudomorphic transformed materials

11. Solid-state NMR spectra of pseudomorphic transformed materials containing surfactant

1. Synthesis of 1,4-((*E*)-2'-Bis(triethoxysilyl)vinyl)benzene (BTEVB)

Chemicals from commercial suppliers: Bis(tri-tert-butylphosphine)palladium(0) (*abcr*, 98 %), 1,4-dibromobenzene (*Aldrich*, 98 %), *N*,*N*-dicyclohexylmethylamine (*Aldrich*, 97 %), triethoxy-vinylsilane (97 %*Merck*).

The reaction was carried out under dry conditions and a nitrogen atmosphere.

1,4-Dioxane was degassed by the freeze-pump-thaw method three times before *N*,*N*-dicyclohexylmethylamine (29 mL, 131 mmol, 2.1 equiv.), triethoxyvinylsilane (27 mL, 131 mmol, 2.1 equiv.) and 1,4-dibromobenzene (15 g, 62 mmol, 1.0 equiv.) were dissolved. The catalyst $Pt[P(^tBu)_3)]_2$ (0.5 g, 0.1 mmol, 0.007 equiv.) was added and the suspension was heated to 80 °C for 18 hours. After quantitative consumption of the starting material the reaction mixture was cooled in an ice bath to precipitate the formed salt quantitatively. The reaction mixture was filtered and washed with *n*-hexane. The filtrate was concentrated under reduced pressure and the residue was diluted in cold *n*-hexane. The organic phase was washed with cold hydrochloric acid (2 x 30 mL, c = 1.0 $mol{\cdot}L^{-1}$) and cold brine (30 mL) quickly, then dried over anhydrous magnesium sulfate. The solvent was removed under vacuum and the raw product was purified by distillation under vacuum. Yield: 21 g (45 mmol, 72 %), colorless liquid

^{1}H-NMR (300 MHz, $CDCl_3$, 25 °C): δ [ppm] = 7.45 (s, 4 H, H2); 7.19 (d, 2 H, ^3J = 19.3 Hz Si-CH=C**H**); 6.18 (d, 2 H, ^3J = 19.3 Hz, Si-C**H**=CH); 3.88 (q, 12 H, $^3J_{CH_2\text{-}CH_3}$ = 7 Hz,C**H₂**); 1.27 (t, 18 H, $^3J_{CH_2\text{-}CH_3}$ = 7 Hz, C**H₃**).

^{13}C {^{1}H}-NMR ($CDCl_3$, 100 MHz, 25 °C): δ (ppm) = 148.6 (Si-CH=**C**H); 138.1 ($C_{q,ar}$); 127.2 (C_{ar}H); 118.4 (Si-**C**H=CH); 58.8 (CH_2); 18.4 (CH_3).

2. SEM images of initial CPG batch

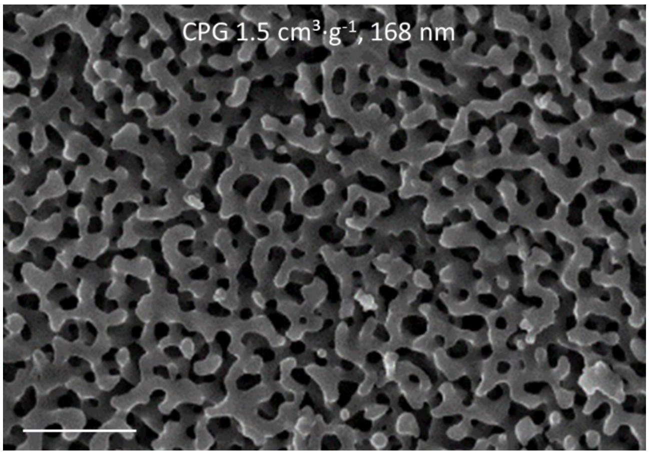

Figure S1: SEM image of the initial CPG batch (magnification: 5×10^4, scale bar: 1 µm).

3. MIP data and calculation of the filling degree

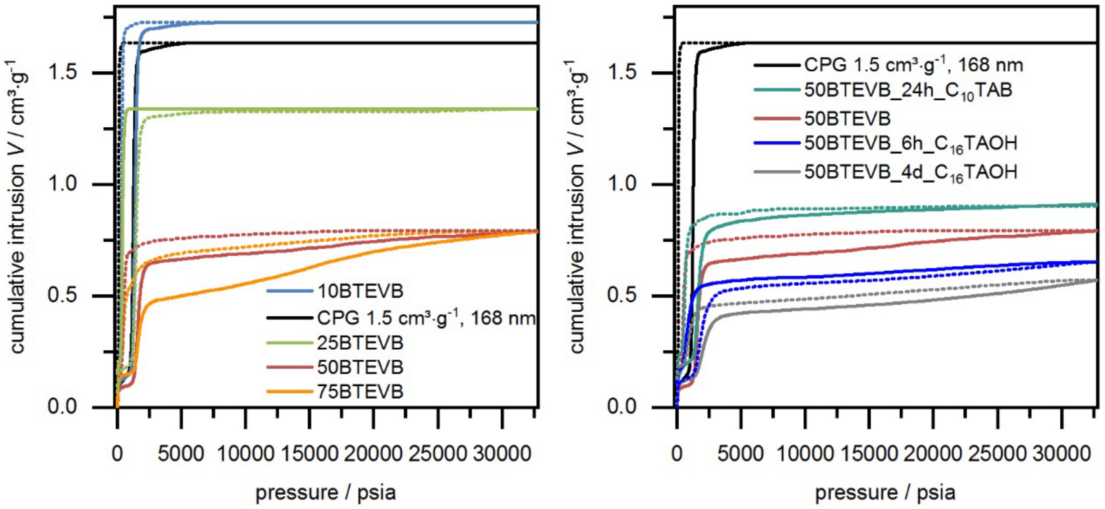

Figure S2: Mercury intrusion curves (extrusion dotted lines) of impregnated samples 10–75BTEVB (left) and 50BTEVB after pseudomorphic transformation (right).

The filling degree is calculated from the intruded pore volume, considering the additional mass of the organosilica phase, which is assumed impenetrable to mercury. The resulting pore volume has to be multiplied with a correction factor as shown in equation (1). The mass of the CPG, the mass of the impregnated hybrid material and the intruded volume are given in Table S1.

$$V_{\text{intr. Hg}} \cdot m_{\text{CPG+Organosilica}} = V_{\text{filled CPG}} \cdot m_{\text{CPG}} \quad (7)$$

$$V_{\text{filled CPG}} = \frac{m_{\text{CPG+Organosilica}}}{m_{\text{CPG}}} \cdot V_{\text{intr. Hg}}$$

where

$V_{\text{intr,Hg}}$	measured mercury intruded volume of the impregnated sample
$m_{\text{CPG+Organosilica}}$	mass of the hybrid material that was measured
m_{CPG}	initial mass of CPG
$V_{\text{filled CPG}}$	real pore volume of the impregnated CPG

With this, the pore volume of filled CPG ($V_{\text{filled CPG}}$) and the volume of the filling (V_{filling}) can be calculated (see equation (2)). The quotient of the filled volume and the initial volume of the CPG batch is the filling degree F as shown in equation (3).

$$V_{\text{filling}} = V_{\text{CPG}} - V_{\text{filled CPG}} \quad (2)$$

$$F = \frac{V_{\text{filling}}}{V_{\text{CPG}}} \quad (3)$$

where

V_{filling}	volume of the filling
V_{CPG}	pore volume of initial CPG batch
F	filling degree

Table S1: Mass of CPG before and after impregnation as used for the calculation of the filling degree, relative pressure range for the determination of the apparent BET specific surface area

	$V_{intr. Hg}$ /cm³·g⁻¹	m_{CPG} / mg	$m_{CPG+organosilica}$ / mg
CPG	1.50		
10BTEVB	1.57	1000	1053
25BTEVB	1.14	1000	1044
50BTEVB	0.69	1995	2856
75BTEVB	0.64	1000	1559

4. Treatment of CPG or BTEVB with ethanol/ sodium hydroxide solution

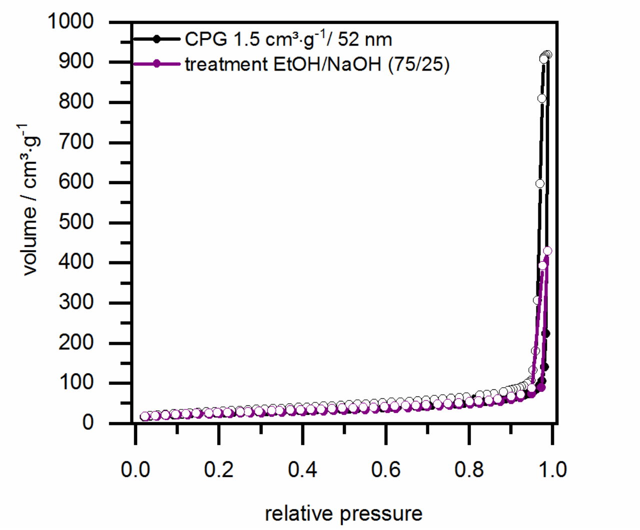

Figure S3: N_2-physisorption isotherm (77 K) of CPG batch (1.5 cm³·g⁻¹, 52 nm) before and after treatment with ethanol/ sodium hydroxide solution.

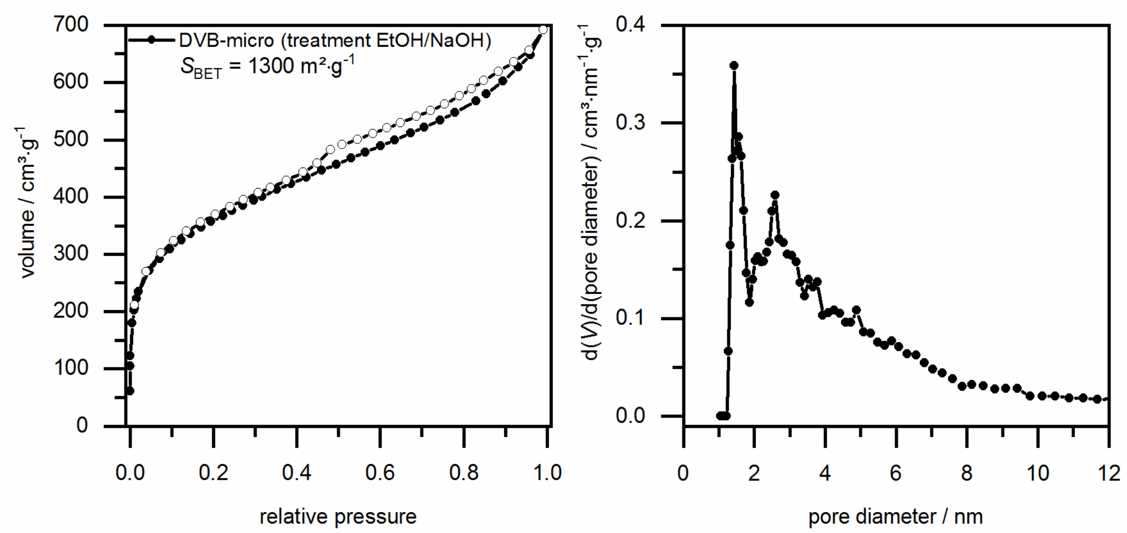

Figure S4: N_2-physisorption isotherm (77 K) of DVB-micro (left) and the pore diameter distribution (NLDFT kernel from adsorption branch for silica with cylindrical pores). DVB-micro represents divinylbenzene-bridged organosilica, which is prepared by treatment of the BTEVB precursor with ethanol / sodium hydroxide solution at 80 °C.

5. TGA of organosilica/silica hybrid materials 10–75BTEVB

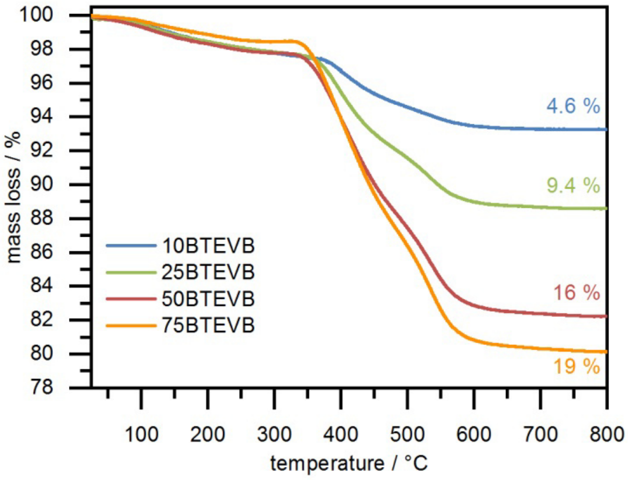

Figure S5: TGA plots of organosilica/silica hybrid materials 10–75BTEVB.

6. TEM images of the initial CPG and 50BTEVB

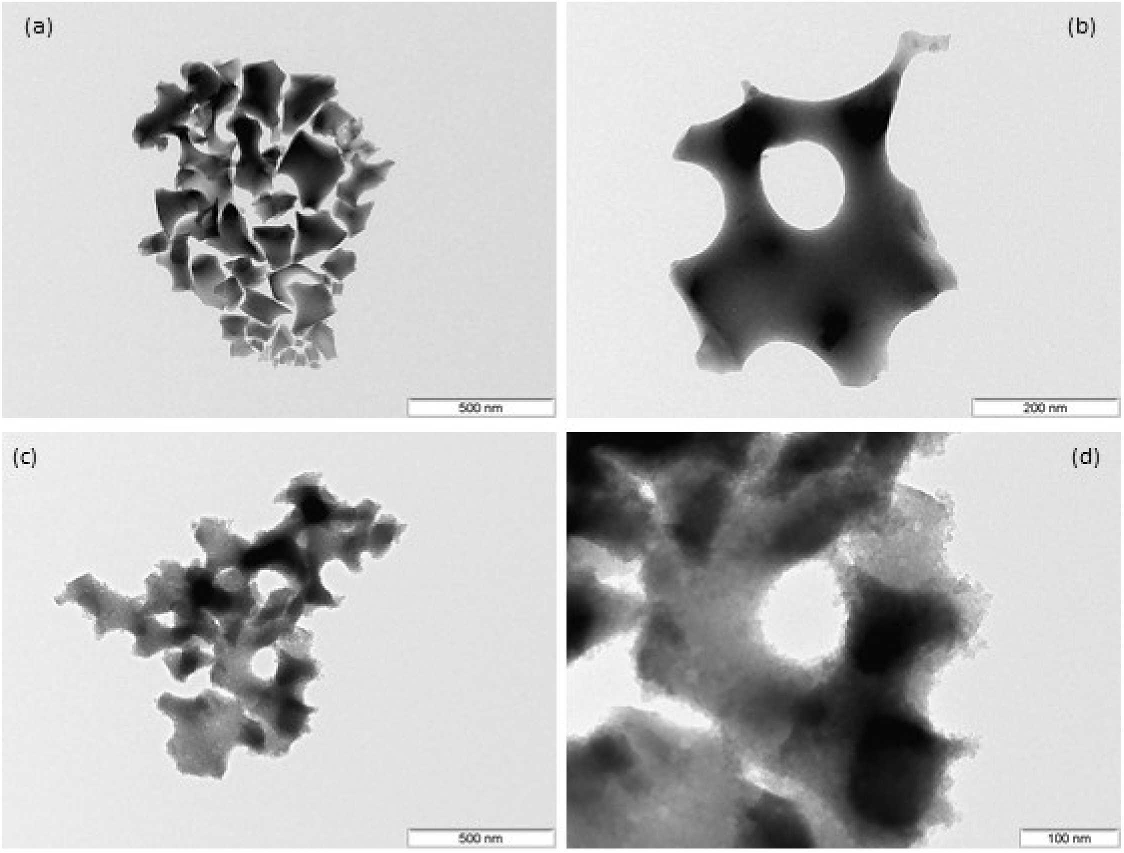

Figure S6: TEM images with different magnification of ground fragments of the initial CPG (a,b) and 50BTEVB (c,d). Scale bars are given in the images.

7. Specific surface area from combined isotherms of type Ib and IVb isotherm

The shape of the isotherm of the sample 50BTEVB_24h_C_{10}TAB makes the determination of the apparent BET specific surface area challenging, since the micropore filling is directly followed by the pore condensation and hence a linear range for the classical BET approach is hard to be determined. However, following the recommendations from IUPAC, the most reasonable result of $S_{BET} = 668\ m^2 \cdot g^{-1}$ is obtained considering the relative pressure range of $p/p^0 = 0.045–0.099$.[2]

8. Solid-state NMR spectra of pseudomorphic transformed materials

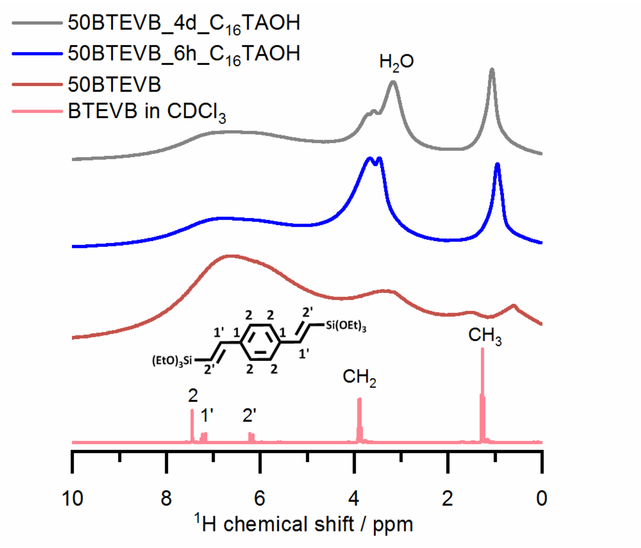

Figure S7: ^{1}H MAS NMR spectra of 50BTEVB, 50BTEVB_4d_C_{16}TAOH and 50BTEVB_6h_ C_{16}TAOH in comparison to the liquid state NMR of BTEVB ($CHCl_3$ at 77.16 ppm).

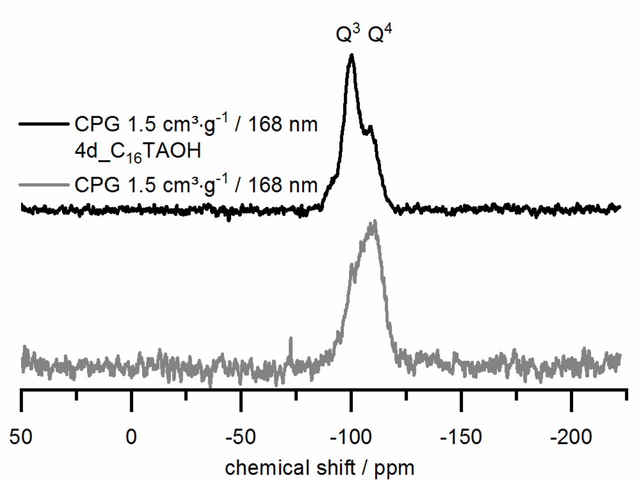

Figure S8: ^{29}Si CP MAS NMR spectra of CPG before (gray) and after four days pseudomorphic transformation with C_{16}TAOH (black).

9. TG/MS data after pseudomorphic transformation

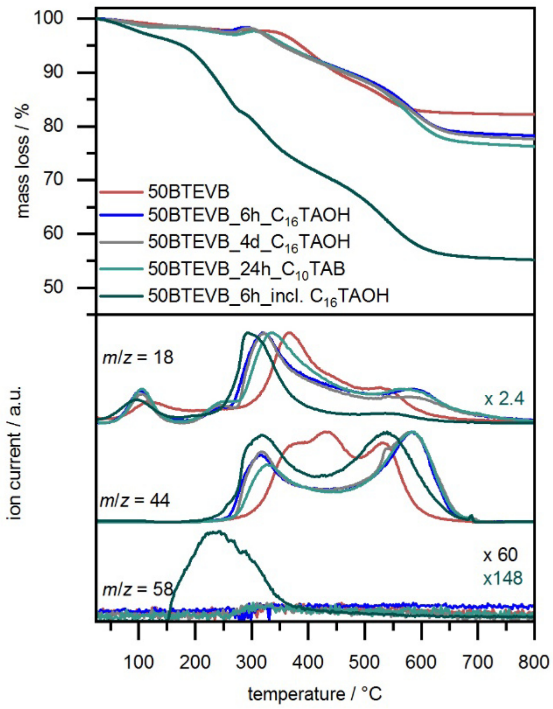

Figure S9: TG/MS analysis in Ar/O_2 80/20 of denoted samples. In the upper plot: mass losses plotted against temperature, lower plot selected ion currents (normalization factors given on the side.)

Table S2: TG mass loss per temperature intervals

	150–300 °C	300–800 °C
**50BTEVB**	1.0 %	16 %
**50BTEVB_6h_C_{16}TAOH**	1.4 %	20 %
**50BTEVB_6h_ incl C_{16}TAOH**	16 %	26 %
**50BTEVB_4d_C_{16}TAOH**	1.4 %	20 %
**50BTEVB_24h_C_{10}TAB**	0.7 %	21 %

Mass loss due to adsorbed water is expected in the temperature interval 25–150 °C. This is corrected in the mass loss

Typical decomposition products of organic compounds, m/z = 44 (CO_2), and water m/z = 18 (H_2O), were tracked. For the detection of the surfactant the ion current of m/z = 58 (C_3H_8N), which is the main fragment in electron impact ionization of quaternary amines, was used.[1]

10. Spectral deconvolution of ^{29}Si MAS NMR of pseudomorphic transformed materials

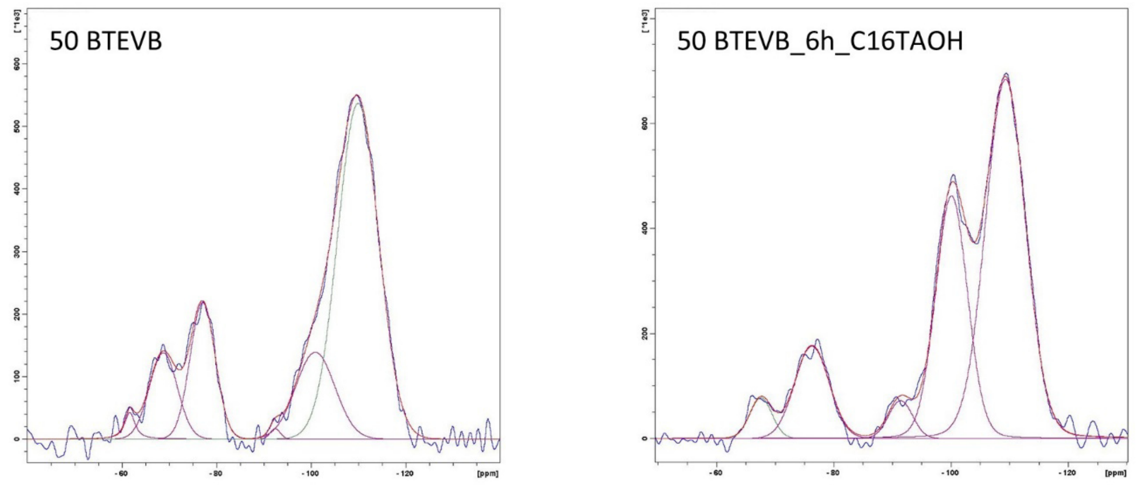

Figure S10: Direct excitation ^{29}Si MAS NMR spectra of 50BTEVB and 50BTEVB_6h_C_{16}TAOH together with spectral deconvolution.

Table S3. Results from quantitative ^{29}Si MAS NMR of 50 BTEVB before and after transformation and resulting organosilica content.

	50BTEVB	50BTEVB_6h_CTAOH
**T^1**	2.8 %	-
**T^2**	18 %	6.1 %
**T^3**	25 %	21 %
**Q^2**	0.77 %	6.1 %
**Q^3**	25 %	54 %
**Q^4**	100 %	100 %
**T**	27 %	15 %
**Q**	73 %	85 %
**mol% organosilica[a]**	16 %	8.1 %

[a] To calculate the molar percentage of organosilica in the hybrid materials, bis-silylated structure of DVB-bridged species is considered.

11. Solid-state NMR spectra of pseudomorphic transformed materials containing surfactant

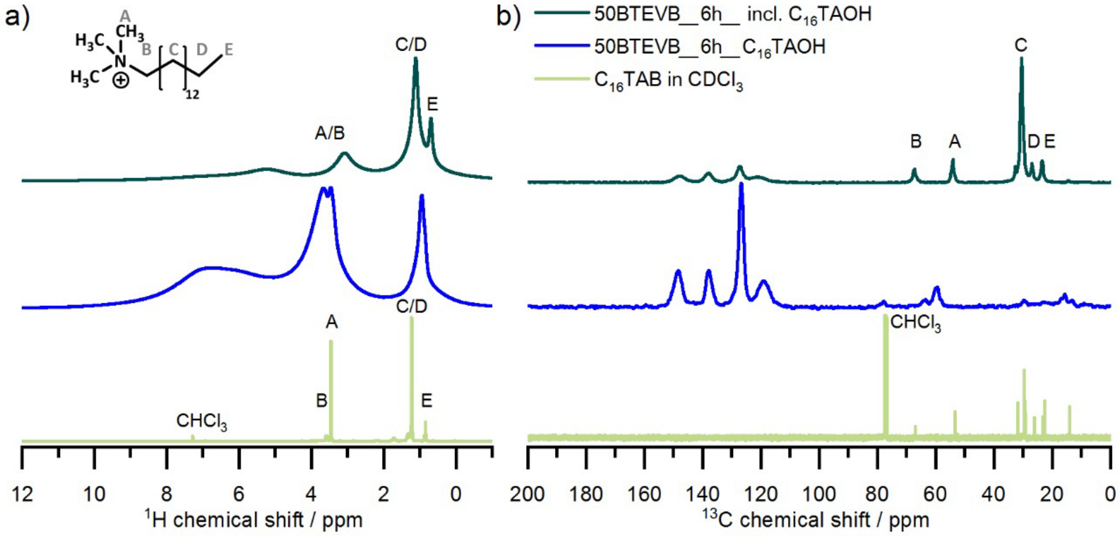

Figure S11: (a) ^{1}H CP MAS NMR spectra of 50BTEVB_6h_C$_{16}$TAOH and 50BTEVB_6h_incl. C$_{16}$TAOH in comparison to the liquid state NMR of C$_{16}$TAB ($CHCl_3$ at 77.16 ppm). (b) ^{13}C CP MAS NMR spectra of these samples.

Bibliography

(1) M. Hesse, H. Meier and B. Zeeh, *Spektroskopische Methoden in Der Organischen Chemie*, 7th editio.; Georg Thieme Verlag: Stuttgart, Germany, **2005**.

(2) M. Thommes, K. Kaneko, A. V. Neimark, J. P. Olivier, F. Rodriguez-Reinoso, J. Rouquerol and K. S. W. Sing, *Pure Appl. Chem.,* **2015**, 87, 1051–1069.
